# Proof of principle study of a detailed whole-body image analysis technique, “Imiomics”, regarding adipose and lean tissue distribution

**DOI:** 10.1038/s41598-019-43690-w

**Published:** 2019-05-14

**Authors:** Lars Lind, Joel Kullberg, Håkan Ahlström, Karl Michaëlsson, Robin Strand

**Affiliations:** 1 Department of Medical Sciences, Uppsala University, Uppsala, Sweden; 2 Division of Radiology, Department of Surgical Sciences, Uppsala University, Uppsala, Sweden; 3 Antaros Medical, BioVenture Hub, Mölndal, Sweden; 4 Department of Surgical Sciences, Uppsala University, Uppsala, Sweden

**Keywords:** Epidemiology, Computational science

## Abstract

This “proof-of-principle” study evaluates if the recently presented “Imiomics” technique could visualize how fat and lean tissue mass are associated with local tissue volume and fat content at high/unprecedented resolution. A whole-body quantitative water-fat MRI scan was performed in 159 men and 167 women aged 50 in the population-based POEM study. Total fat and lean mass were measured by DXA. Fat content was measured by the water-fat MRI. Fat mass and distribution measures were associated to the detailed differences in tissue volume and fat concentration throughout the body using Imiomics. Fat mass was positively correlated (r > 0.50, p < 0.05) with tissue volume in all subcutaneous areas of the body, as well as volumes of the liver, intraperitoneal fat, retroperitoneal fat and perirenal fat, but negatively to lung volume. Fat mass correlated positively with volumes of paravertebral muscles, and muscles in the ventral part of the thigh and lower limb. Fat mass was distinctly correlated with the fat content in subcutaneous adipose tissue at the trunk. Lean mass was positively related to the large skeletal muscles and the skeleton. The present study indicates the Imiomics technique to be suitable for studies of fat and lean tissue distribution, and feasible for large scale studies.

## Introduction

Both the amount of adipose tissue and the distribution are major characteristics to be taken into account in obesity. It is known from previous imaging studies that subjects with a high waist/hip-ratio (WHR) have increased amounts of fat in predefined ectopic fat depots, like the liver, intraperitoneally, retroperitoneally, perirenal and in the epi/pericardium^1–4^, but the exact anatomical correlates of an increased fat mass or a high WHR are not known in detail. Another important characteristic of body composition is lean mass, which mainly constitutes of skeletal muscle mass.

We have recently developed a new way to analyze whole-body MRI data in which the volume and fat content (percentage of fat) of the more than two million image elements included in a whole-body scan are quantified against a reference human (one male and one female), so called “Imiomics^5^”. By doing so, it is possible to correlate any phenotype vs each image element in terms of expansion/contraction and fat content and anatomical correlation maps could be constructed. This will create a high-resolution anatomical view on the correlation vs the phenotype, without the need for predefined fat depots or their explicit quantifications.

The Imiomics technique has previously been described and evaluated in detail^5^. It has been demonstrated robust in applications in a large cohort, and to give an accuracy evaluation score denoted inverse consistency of less than 5 mm, when averaged over the whole body.

The untargeted and highly spatially detailed Imiomics analyses can, as opposed to traditional techniques based on analysis in predefined regions-of-interest (ROIs), capture, for example, intra-organ variations due to heterogeneous tissue or findings in organs where relations could not be expected. Imiomics is thus a technique with the potential to find new relations between non-imaging parameters and MRI imaging data (tissue, organs, or parts of organs). The technique enables findings both on the organ and tissue level, and on the voxel-level, i.e., within organs and tissue, which fundamentally differs from conventional analyses based on pre-defined regions of interest.

In the present study, we used the Imiomics technique to relate the two major characteristics of obesity, fat mass and WHR, to local tissue volume and fat content in the whole body in a population-based sample of 326 subjects aged 50 in the Prospective study on Obesity, Energy, and Metabolism (POEM) study^6^. The hypothesis tested in this “proof-of principle” study was that we by image analysis could visualize how an increased fat mass and a high WHR would influence the volume and fat content of different parts of the body with a high degree of resolution. In addition, we investigated the anatomical correlate to lean mass using Imiomics.

## Materials and Methods

### Sample

The Prospective study on Obesity, Energy, and Metabolism (POEM) recruited males and females aged 50 years from the general population by a random invitation by mail using official registers of the inhabitants of the city of Uppsala, Sweden^6^. The participants received an invitation one month following their 50^th^ birthday. In total, 502 individuals participated in the study with a participation rate of 25%. Since the participants were invited randomly from the population, not everyone was completely healthy. In this sample, 2.0% had known diabetes, 8,4% had known hypertension, 3.8% were on statin treatment, but no one suffered from severe diseases, such as cancer, myocardial infarction, stroke, heart failure or chronic obstructive lung disease. The study was approved by the ethics committee at Uppsala University (Approval numbers: Uppsala Dnr 2009/057 and Dnr 2012/143), and the participants gave their informed consent. The research was performed in accordance with relevant guidelines and regulations.

The participants were investigated after an overnight fast. Waist and hip circumference were measured at the umbilical and trochanter levels, respectively and WHR was calculated. Fat and lean mass was measured by DXA. Whole-body MRI was performed within one month at a separate day.

### DXA

Total and regional body fat and lean mass were estimated using Dual-energy X-ray absorptiometry (DXA; Lunar Prodigy, GE Healthcare). In order to minimize the potential operator bias all scans were performed in the same room by one experienced nurse. By triple measurements in 15 subjects with repositioning according to recommendations from the International Society for Clinical Densitometry, the precision error of the DXA measurements in our laboratory has been calculated. Total fat and lean mass had a precision error of 1.5% and 1.0%, respectively. The long-term coefficient of variation was <1% for a spine phantom^7^. For analysis, the automatic edge detection was always used; however, all scans were thoroughly checked for errors and manually corrected if needed.

### MRI

All subjects were imaged on a 1.5T clinical MR system (Philips Achieva, Philips Healthcare, Best, Netherlands) in supine position using a continuously moving bed setup and the body coil. Imaging included a whole body water-fat imaging protocol that used a spoiled 3D multi gradient echo sequence. Scan parameters were: TR/TE1/ΔTE = 5.9/1.36/1.87 ms, 3 unipolar echoes, flip angle 3 degrees. Imaged field of view (FOV) 530 × 377 × 2000 mm^3^, reconstructed voxel size 2.07 × 2.07 × 8.0 mm^3^ in left-right × anterior-posterior × foot-head directions. The scan time was 5 min 15 s. See Kullberg *et al*.^8^ for acquisition details.

Water-fat image reconstruction, which gives voxel-wise fat content quantification, was performed using an in-house developed algorithm.

The imaging protocol and the in-house developed water-fat image reconstruction gives fewer fat/water swaps compared to the MR system’s built-in reconstruction and have previously been described^9^.

### Imiomics

A key component of the Imiomics technique is image registration. In image registration, a target image is deformed to match a reference (fixed) image by computing and applying a deformation field^5^. The deformation field defines for each point in the reference image a corresponding point in the target image. These point-by-point correspondences are utilized in Imiomics analyses, where whole body MRI images are deformed to a reference whole body MRI volume. The reference whole body MRI volume constitutes a reference coordinate system, in which each point has a corresponding point in all volumes in the cohort.

The Imiomics image registration method utilizes a tissue-specific handling of bone, lean tissue, and adipose tissue. See^5^ for a more detailed description of the pipeline. The degree of elasticity of the deformation required to align two images tends to differ between these different tissue types. This prior knowledge is utilized by performing the image registration of the different tissues sequentially, using appropriate registration parameters for each tissue. The Imiomics image registration method consists of the following three steps. (1) Articulated, piece-wise affine, registration of bone sections. (2) Registration of water images with constraints on bone. (3) Registration of fat images with constraints on bone and water. This image registration method has been presented and evaluated^10^ on MRI water-fat image data and achieves image registration results suitable for Imiomics analyses.

**Figure 1:**
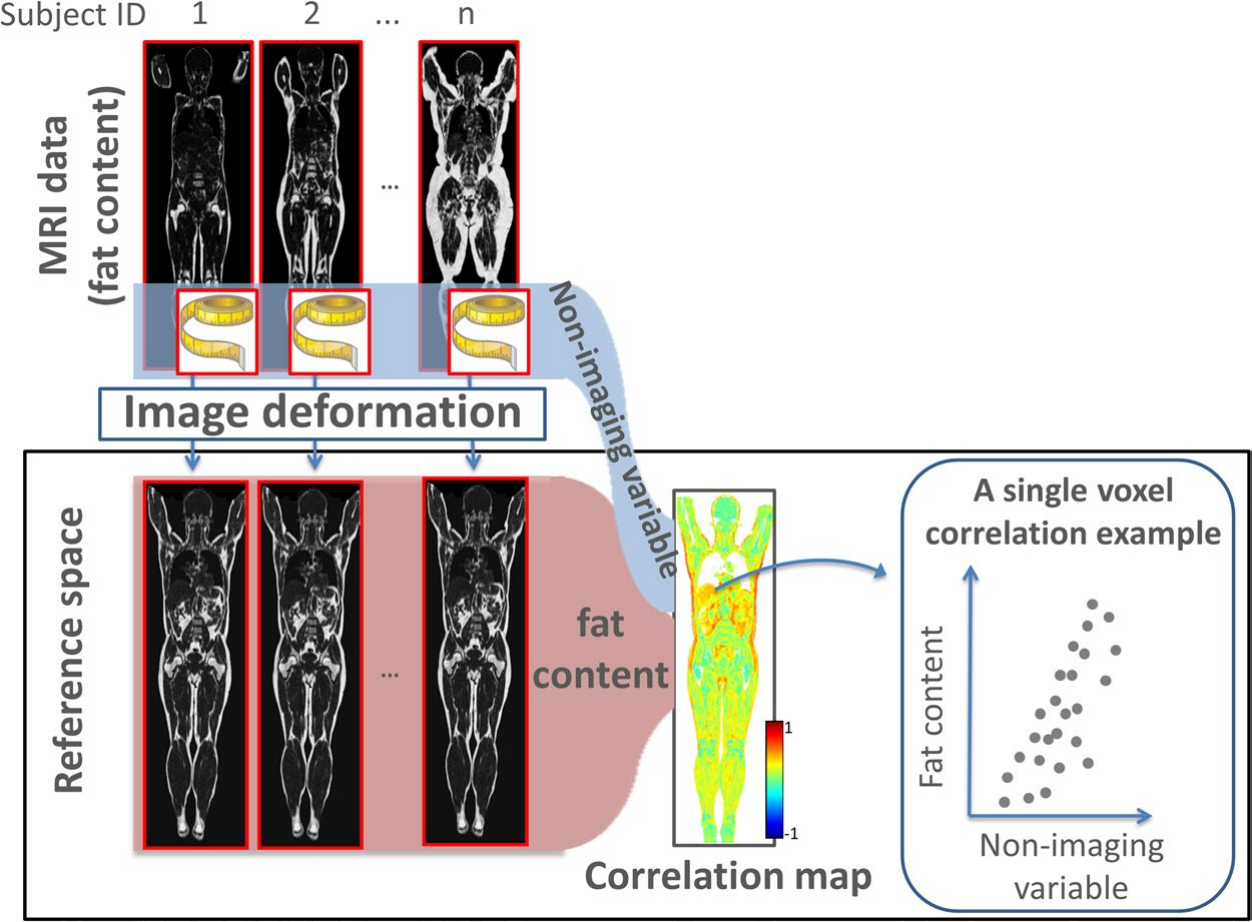
Imiomics analyses. All whole-body MRI volumes are deformed to a reference space by image registration. The point-to-point correspondences in the reference space enable the computation of point-wise correlations. In this conceptual illustration, the correlation between the imaging parameter fat content and the non-imaging parameter total fat mass is analysed. The scatter plot in a single voxel with positive correlation is shown to the right.

In the Imiomics statistical analysis procedure^5^, correlations between imaging and non-imaging parameters are computed in the reference coordinate system. In this study, the global, non-imaging parameters DXA and waist-to-hip-ratio, and the point-wise imaging parameters local tissue volume and fat content were considered. The local tissue volume can be computed from the amount of local expansion and contraction, which is directly given by the deformation field. The Pearson correlation coefficient between the non-imaging parameters and the imaging parameters were computed for each voxel in the reference coordinate system. See Fig. 1 for a conceptual illustration of the Imiomics analysis procedure.

### Statistics

The main analysis in this study was performed using voxel-wise Pearson correlations. To assess the eventual bias from outliers and non-normality also Spearman correlations were calculated.

In the present study, approximately 10^7^ statistical tests were performed. This could potentially create a large number of false positive findings. Therefore, to limit the chance of false positive findings we only report findings that have an absolute correlation coefficient >0.50 (orange to dark red or blue for positive and negative correlations, respectively, in the color coding in the images) in several adjacent image elements that together make up a plausible anatomical structure. Furthermore, we require that the corresponding p-value map to uniformly show p < 0.05 (red to brown) for all image elements in that anatomical structure. In addition, we require that, expect from a more gynoid fat distribution, the findings must be similar in men and women, and that the results should be similar using non-parametric correlation analysis limiting the effect of outliers.

## Results

The anthropometric data and data derived from DXA in the sample are given for males and females in Table 1.

**Table 1:** Mean and SD for anthropometric and fat mass and lean mass obtained at the DXA investigation.

Variable	Women		Men	
	N	Mean (SD)	N	Mean (SD)
Height (cm)	167	166.2 (6.6)	159	179.2 (6.3)
Weight (kg)	167	71.8 (12.6)	159	85.7 (11.9)
BMI (kg/m^2^)	167	26 (4.6)	159	26.7 (3.6)
Waist/hip ratio	167	0.87 (0.07)	159	0.93 (0.06)
Fat mass (kg)	156	26.7 (9.9)	151	22.0 (8.8)
Lean mass (kg)	156	42.3 (4.7)	151	60.2 (5.9)

### Tissue volume associations to fat mass

**Figure 2:**
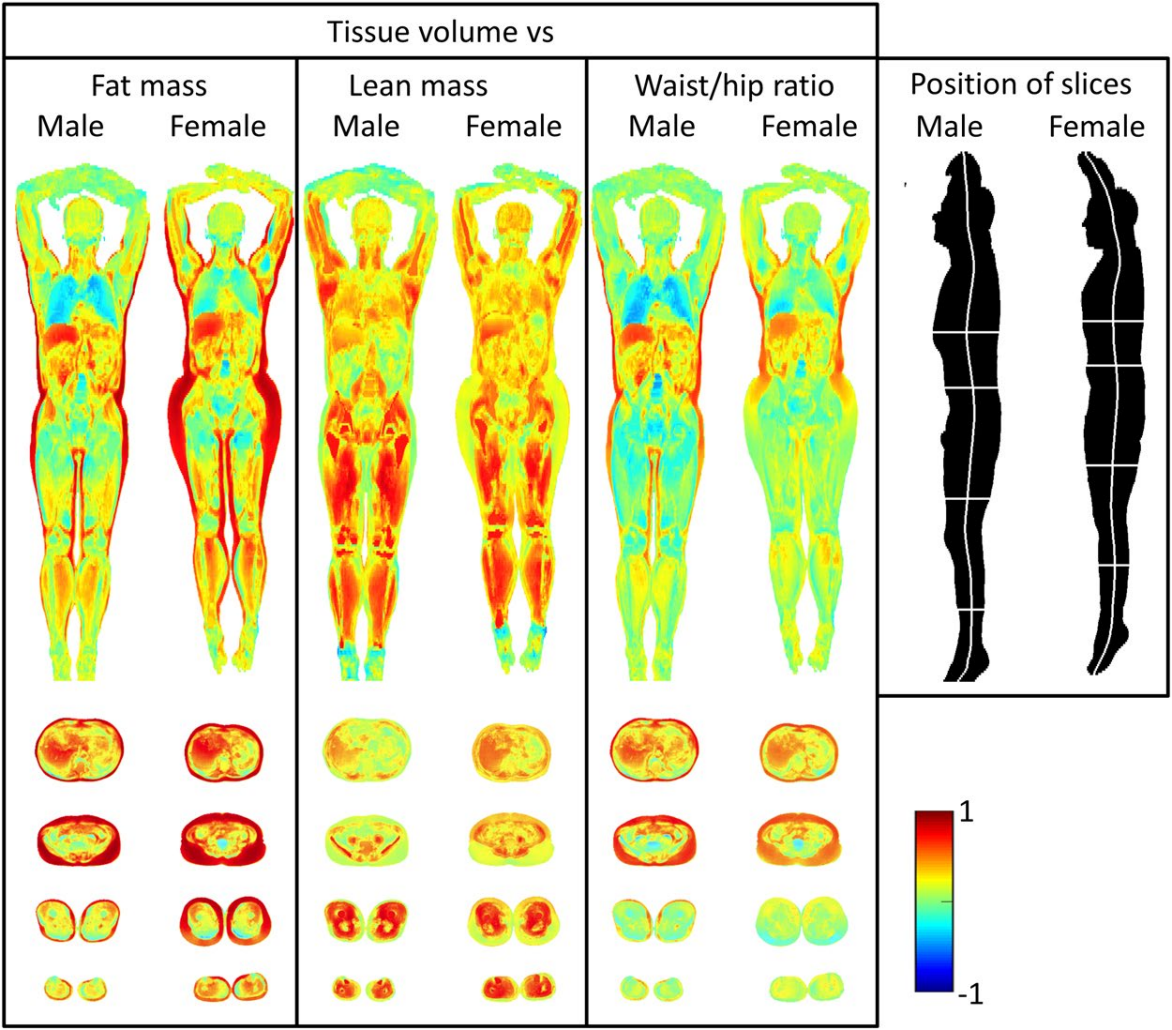
Imiomics analysis results on local tissue volume. Curved coronal and axial slices of the full volume images are shown. Color-coded correlation values (r, Pearson coefficient) of non-imaging parameters total fat mass (measured by DXA, left), total lean mass (measured by DXA, middle), and waist/hip ratio (right) vs, local tissue volume (Jacobian determinant of the deformation field).

As could be seen in the coronal images in Fig. 2, in women, fat mass was correlated positively with local tissue volume especially in the hip region, but also in all subcutaneous areas of the body. In addition, fat mass was correlated with volumes of the liver and perirenal fat, but on the contrary fat mass was inversely correlated with volumes of the lungs, lumbar vertebraes of the spine and the femur. Transversal images also disclose positive correlations with intraperitoneal fat, retroperitoneal fat and muscles in the thigh and lower limb.

A similar picture was seen in men.

### Tissue volume associations to lean mass

As could be seen in the coronal images in Fig. 2, in women, lean mass is associated with the local volumes of large muscles in the legs and arms. Lean mass is also related to the local volumes of the long bones in the arm and the legs. Lean mass also tended to be related to the local volume of the lungs and the liver. A similar picture emerges in males, but is even more distinct than in women regarding the local volumes of large muscles in the legs and arms. Correlations are also seen with the psoas muscle and the pelvic bone

### Tissue volume associations to WHR

In females, WHR was correlated (r > 0.50, p < 0.05) with larger volumes in the superficial subcutaneous regions of the upper parts, but not lower parts of the body. WHR was furthermore positively correlated with volumes of the liver and perirenal fat, but negatively with the volume of the lungs. Transversal images showed positive correlations with intraabdominal fat and retroperitoneal fat, but in this case not with muscles in the thigh and lower limb. A similar picture was seen in men See Fig. 2.

It should be pointed out that the correlations between WHR and fat depots generally were weaker than those found for fat mass.

### Fat content associations to fat mass

**Figure 3:**
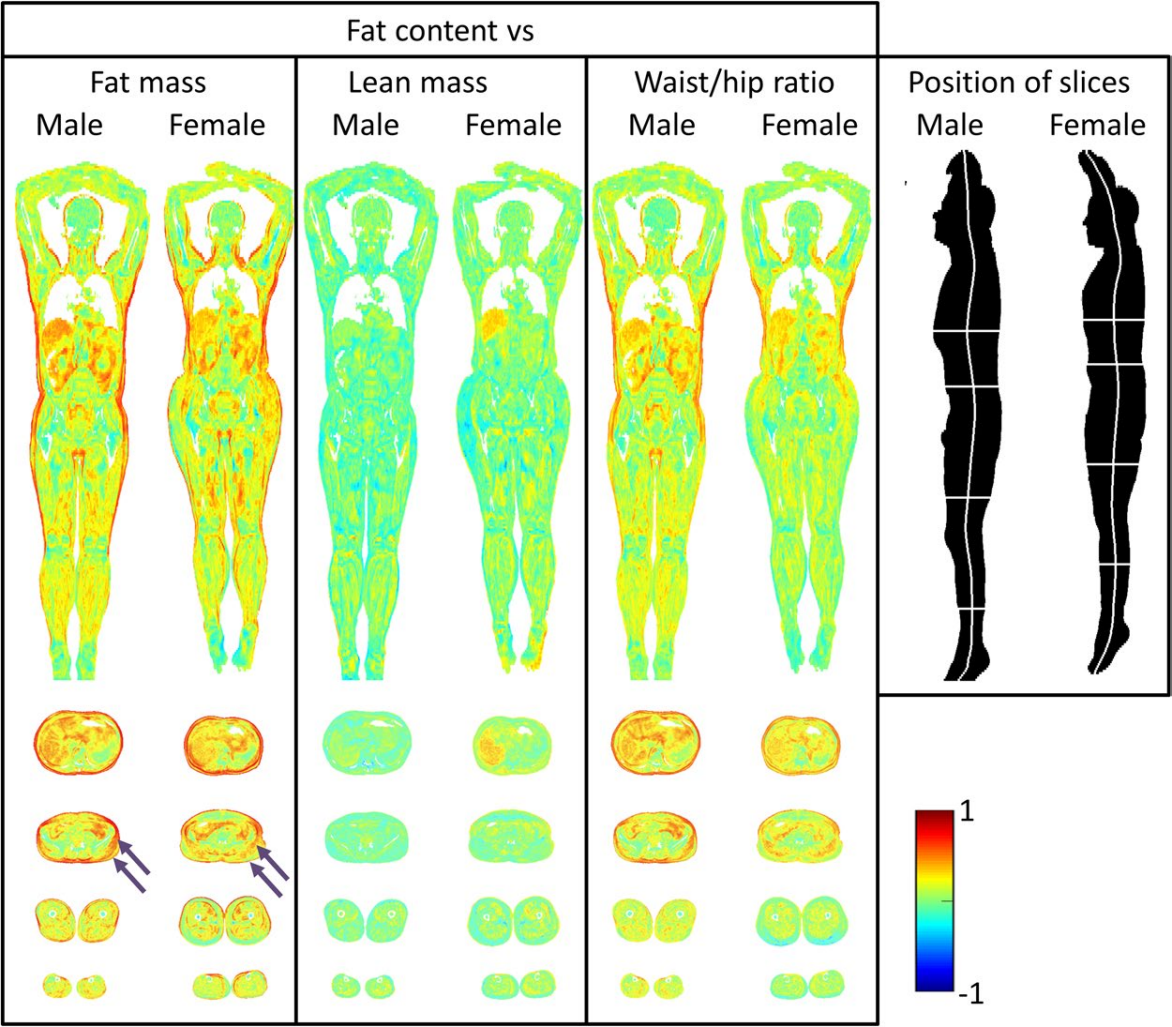
Imiomics analysis results on local fat content. Curved coronal and axial slices of the full volume images are shown. Color-coded correlation values (r, Pearson coefficient) of non-imaging parameters total fat mass (measured by DXA, left), total lean mass (measured by DXA, middle), and waist/hip ratio (right) vs fat content (from quantitative MR images). The purple arrows indicate where there is a positive relation to two distinct layers in the subcutaneous adipose tissue at the trunk.

Fat mass was correlated only with the fat content in a thin layer of the superficial subcutaneous areas of the body in women. See Fig. 3. In addition, fat mass also was correlated with a deeper part of the subcutaneous tissue, which could be seen both on the coronal and transversal images. Fat mass was also correlated with the fat content of perirenal fat and intraabdominal fat, but less so vs. the fat content of the liver. A similar picture was seen in men.

### Fat content associations to WHR

WHR was correlated only with the fat content in a thin layer of the superficial subcutaneous areas as well in a deeper depot in the adipose tissue in women, but in this case only in the upper part of the body. See Fig. 3. The correlations vs fat content in perirenal fat, intraabdominal fat and in the liver was less evident for WHR compared to fat mass.

A similar picture emerged in men, although the relationships between WHR and fat content in the adipose tissue depots seemed to be generally stronger than in the women.

### Fat content associations to lean mass

Lean mass was not related to fat content in any part of the body in a powerful manner. See Fig. 3.

## Discussion

As expected, the Imiomics technique showed that an increase in fat mass was positively correlated with volume of subcutaneous fat, as well as in other fat depots, such as liver, perirenal fat, intra-peritoneal fat, and retroperitoneal fat. An increase in WHR, on the other hand, was related to larger volumes of fat depots mainly in the upper parts of the body.

Similar pictures emerged when the fat content was measured instead of volumes, but in this case the fat content in the subcutaneous adipose tissue could be distinctly divided into a superficial and a deep portion. As expected, lean mass was mainly related to the volumes of the major skeletal muscles. Thus, this proof of principle study shows the Imiomics technique to be suitable for the study of relationships between body composition phenotypes and multiple adipose and lean tissue depots with a high degree of resolution.

In addition to the positive correlations between fat mass and volumes of multiple fat depots, fat mass was also correlated to volumes of paravertebral muscles, and muscles in the thigh and lower limb (especially on the ventral part of the legs). This is likely to reflect the fact that obese subjects demand on an expanded musculature in these regions in order to keep a standing position.

It has been known for a long time that obesity impairs respiratory function with a reduction in vital capacity^11,12^, and the observed negative correlation between an increased fat mass and lung volume is the “imiomic” consequence of such relationship.

When measuring the volumes of the collum/trochanter, the epichondyl regions of the femur and the lumbar vertebraes with MRI, a positive relationship was seen vs lean mass and a negative association vs fat mass. Increased bone volumes are required to meet an increased body mass, but although a positive relationship has been found between fat mass and bone mineral content at DXA in some observational, as well as genetic studies^13^, more fragile bones have been associated with obesity^14^. Thus, the observed negative relationship between bone volume and fat mass is not clear and deserves further investigations.

The subcutaneous fat could be divided into a superficial and a deep part, and these two parts differ in terms of gene expression^15^, where the deep subcutaneous fat more resembles visceral fat. Using quantification of the fat content rather than the volume of the fat depots, fat mass, and to some extent also WHR, was positively related to two distinct layers in the subcutaneous adipose tissue at the trunk (see Figs 2 and 3). The reasons for these associations can only be speculated upon. One possible reason for variations in fat content or concentration in adipose tissue might be adipocyte size. It is known that adipocyte size is associated to BMI and we have also previously seen relatively large variations in fat concentration in subcutaneous adipose tissue^16^. The spatial agreement in the image registration method is high in general^5^, and we can expect the two distinct layers in the subcutaneous adipose tissue at the trunk to agree well between the deformed and reference images. It should however be noted that no care is taken to explicitly attempt to register the facia lata during the Imiomics image registration. This potential method improvement is left for a future study.

### Technical aspects including limitations

A limitation of the current study is the relatively low registration accuracy in feet, the forearm, spine (male) and upper part of liver (male) (see Supplementary Figs S1–S12). Also, when the layer of subcutaneous fat is thin, partial volume effects can influence the fat content in individual voxels and thus effect correlations. The low accuracy in the forearm was due to non-standardized position and limited field-of-view, in the upper part of the liver was mainly due to breathing artefacts and in the spine was due to the difficult pre-segmentation of the spine. The inverse consistency errors are based on the composition of two deformations. The regions in the spine and liver with the largest errors are in the magnitude of two times the resolution in the axial direction. Note that this corresponds to the composition of two deformation fields where each has an error corresponding to the voxel size.

The individuals had at most one month between the DXA and MRI scans. A limitation is that we do not know if any weight change occurred during this period. However, such as change would only drive the correlations towards the null-hypothesis and cannot produce any false positive findings.

The quality of the water and fat reconstructed images might be limited by phase errors due to for example eddy currents^17^ or to the moving bed approach applied. The image data was screened for deficiencies from e.g. implants and non-standardized positioning.

In conclusion, the present “proof-of -principle” study relating fat mass, lean mass and WHR to local tissue volume and fat content at the whole-body level indicates the Imiomics technique to be suitable for the study of relationships between body composition phenotypes and multiple tissues at a high resolution.

## Supplementary information

Detailed correlation maps

## Data Availability

All data generated and analysed during this study are included in this published article (and its Supplementary Information files). The measurements on waist-hip-ratio, total fat mass and total lean mass analysed during the current study are not publicly available, since no informed consent has been given by the participants for making data publically available. The image data is available from lars.lind@medsci.uu.se on reasonable request.
