# Supplementary material for: Proof of principle study of a detailed whole-body image analysis technique, “Imiomics”, regarding adipose and lean tissue distribution
Source: Sci Rep. 2019 May 14;9:7388. doi: 10.1038/s41598-019-43690-w (PMC6517436; doi:10.1038/s41598-019-43690-w)
Supplement: Supplementary file 1 — Detailed correlation maps [file 41598_2019_43690_MOESM1_ESM.docx]

**Supplementary information**

Proof of principle study of a detailed whole-body image analysis technique, “Imiomics”, regarding adipose and lean tissue distribution

Lars Lind^1^, Joel Kullberg^2,3^, Håkan Ahlström^2,3^, Karl Michaëlsson^4^, Robin Strand^2*^

^1^ Department of Medical Sciences, Uppsala University, Uppsala, Sweden

^2^ Division of Radiology, Department of Surgical Sciences, Uppsala University, Uppsala, Sweden

^3^ Antaros Medical, BioVenture Hub, Mölndal, Sweden

^4^ Department of Surgical Sciences, Uppsala University, Uppsala, Sweden

^*^ Corresponding author

Email: Robin.Strand@it.uu.se

**S1 Fig. Imiomics analysis – local tissue volume vs. total fat mass for men.** Imiomics analysis of local tissue volume (from whole body MRI) vs. total fat mass (measured by DXA) for men. This figure gives detailed information to supplement the leftmost subfigures in Fig 2 in the main manuscript. The fat content values are between 0% (black) and 100% (white). The interval to which the colors in the colormap (shown to the right) is mapped to is shown within brackets for p-map, r-map and the displacement error as computed by inverse consistency. The position of the curved coronal and four axial slices are shown in the right panel. N=151.

| Fat | p map | r map | displacement error |  | |
| --- | --- | --- | --- | --- | --- |
| [0 100] | [1 0] (red sign.) | [-1 1] | [0 20] mm |  |  |
| 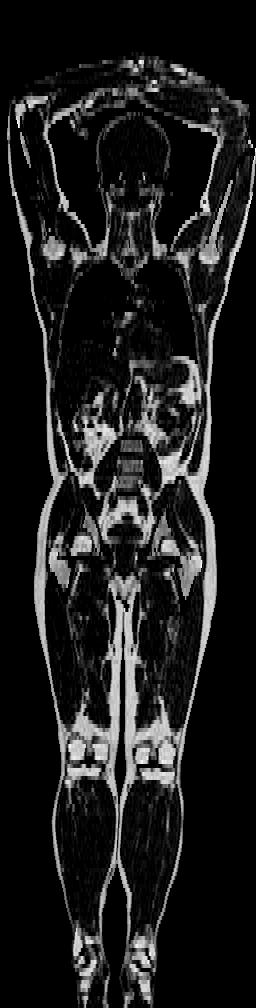 | 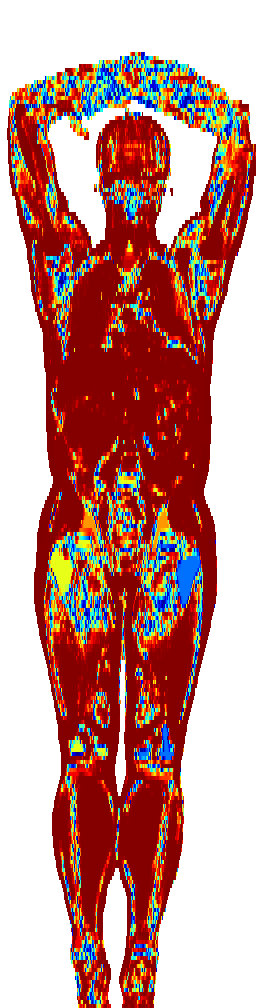 | 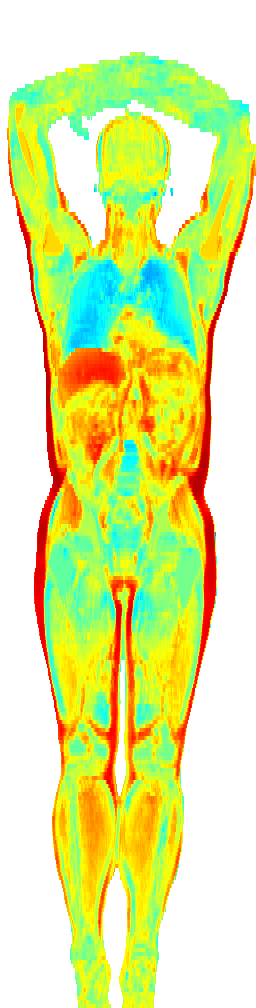 | 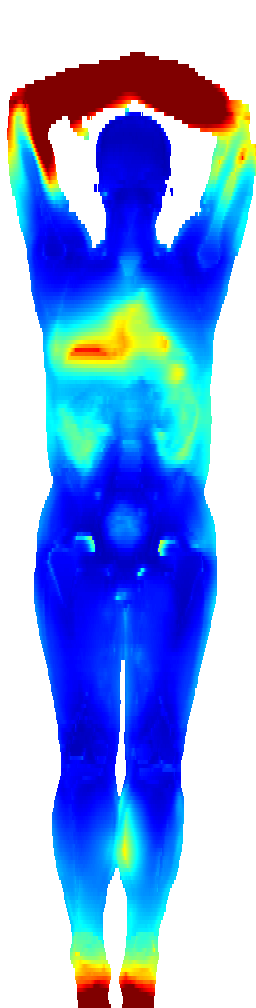 | 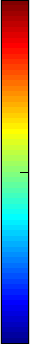 | 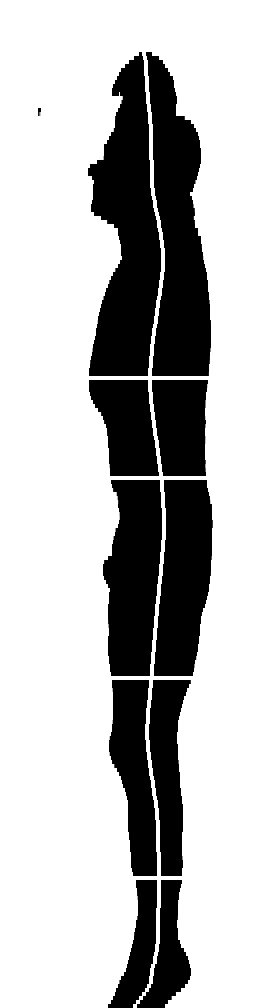 |
| 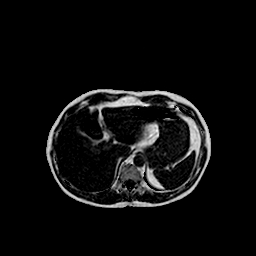 | 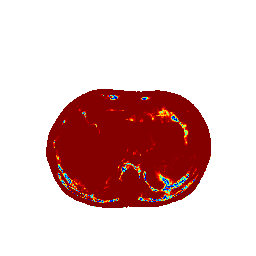 | 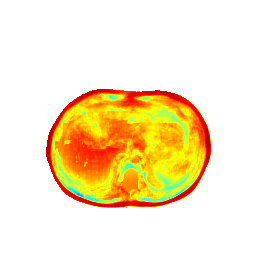 | 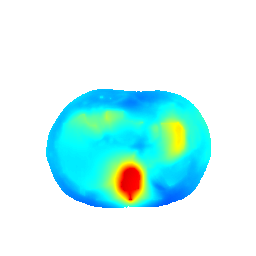 |  | |
| 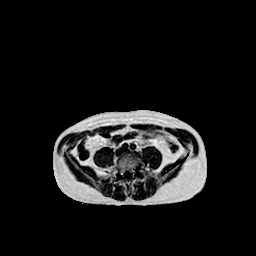 | 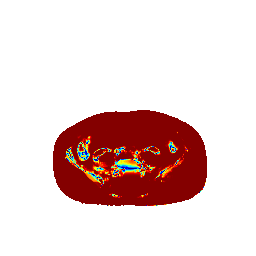 | 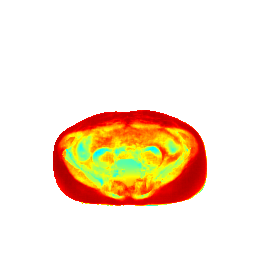 | 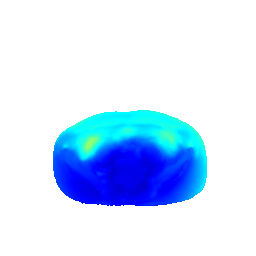 |  |  |
| 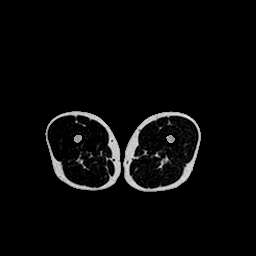 | 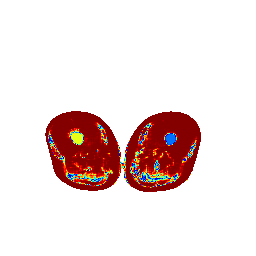 | 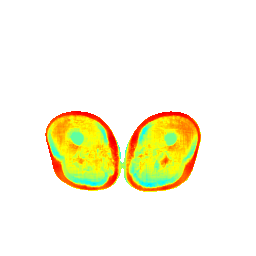 | 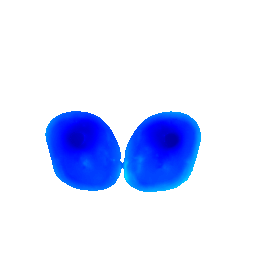 |  |  |
| 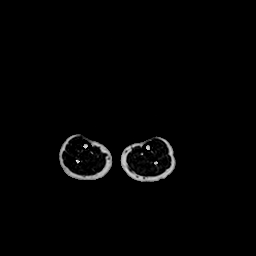 | 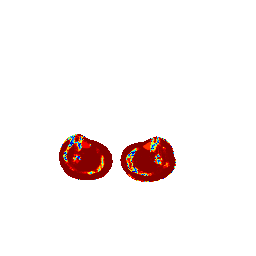 | 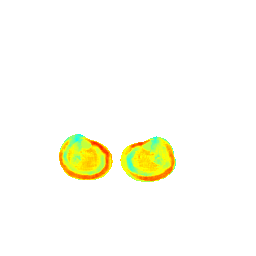 | 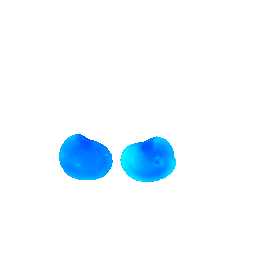 |  |  |
|  |  |  |  |  |  |

**S2 Fig. Imiomics analysis – local tissue volume vs. total fat mass for women.** Imiomics analysis of local tissue volume (from whole body MRI) vs. total fat mass (measured by DXA) for women. This figure gives detailed information to supplement the second subfigure from the left in Fig 2 in the main manuscript. The fat content values are between 0% (black) and 100% (white). The interval to which the colors in the colormap (shown to the right) is mapped to is shown within brackets for p-map, r-map and the displacement error as computed by inverse consistency. The position of the curved coronal and four axial slices are shown in the right panel. N=156.

| Fat | p map | r map | displacement error |  | |
| --- | --- | --- | --- | --- | --- |
| [0 100] | [1 0] (red sign.) | [-1 1] | [0 20] mm |  |  |
| 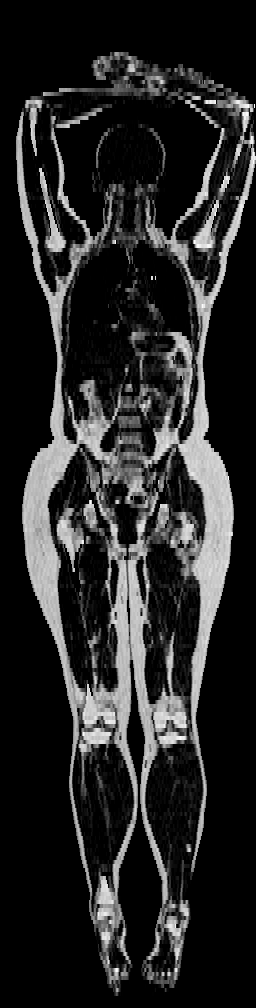 | 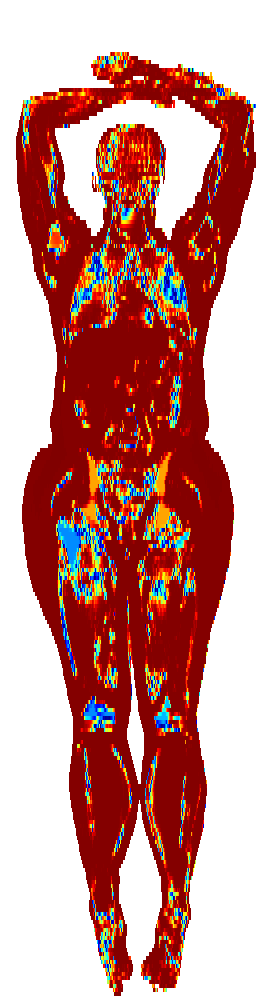 | 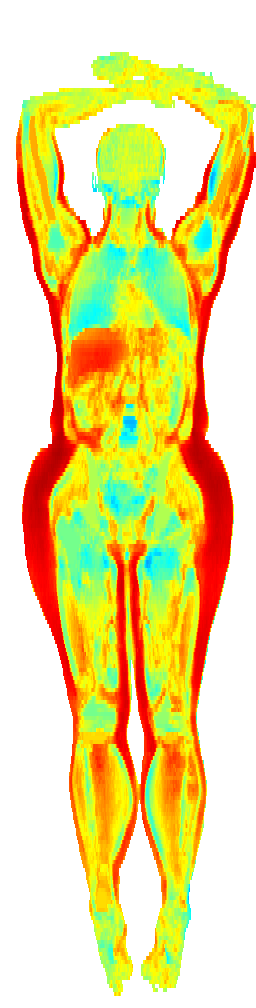 | 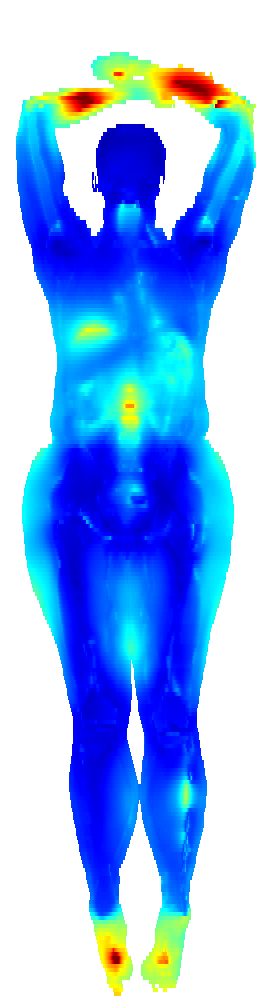 | 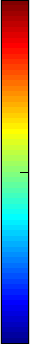 | 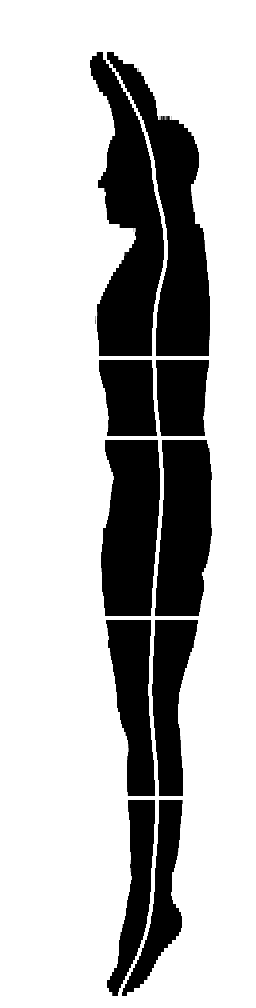 |
| 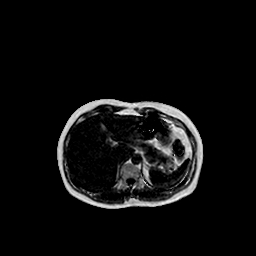 | 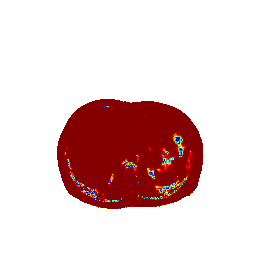 | 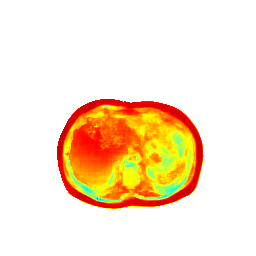 | 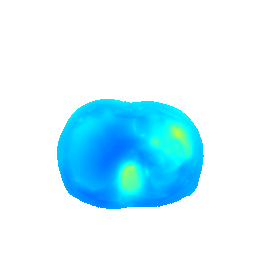 |  | |
| 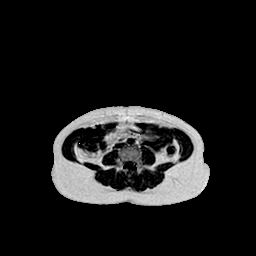 | 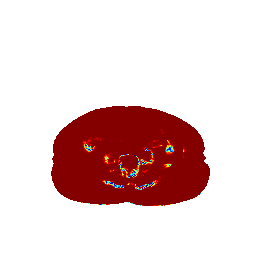 | 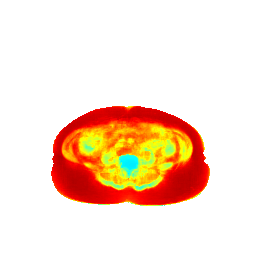 | 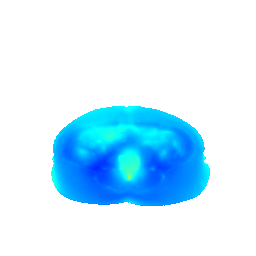 |  |  |
| 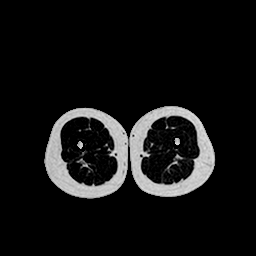 | 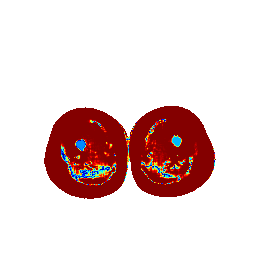 | 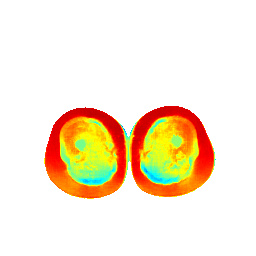 | 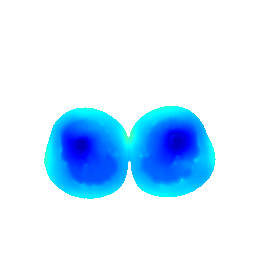 |  |  |
| 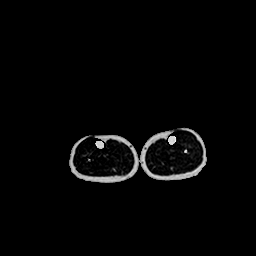 | 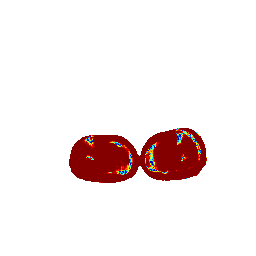 | 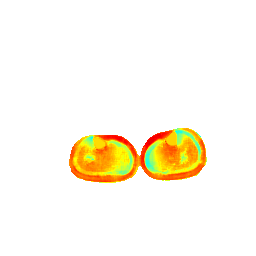 | 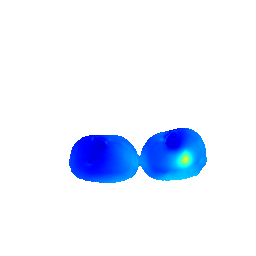 |  |  |
|  |  |  |  |  |  |

**S3 Fig. Imiomics analysis –local tissue volume vs. total lean mass for men.** Imiomics analysis of local tissue volume (from whole body MRI) vs. total lean mass (measured by DXA) for men. This figure gives detailed information to supplement the third subfigures from the left in Fig 2 in the main manuscript. The fat content values are between 0% (black) and 100% (white). The interval to which the colors in the colormap (shown to the right) is mapped to is shown within brackets for p-map, r-map and the displacement error as computed by inverse consistency. The position of the curved coronal and four axial slices are shown in the right panel. N=151.

| Fat | p map | r map | displacement error |  | |
| --- | --- | --- | --- | --- | --- |
| [0 100] | [1 0] (red sign.) | [-1 1] | [0 20] mm |  |  |
| 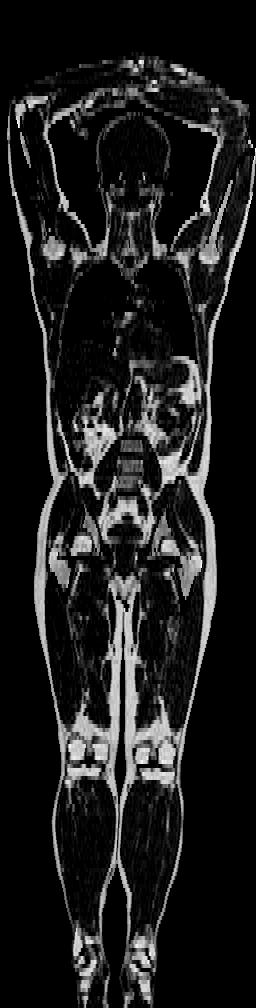 | 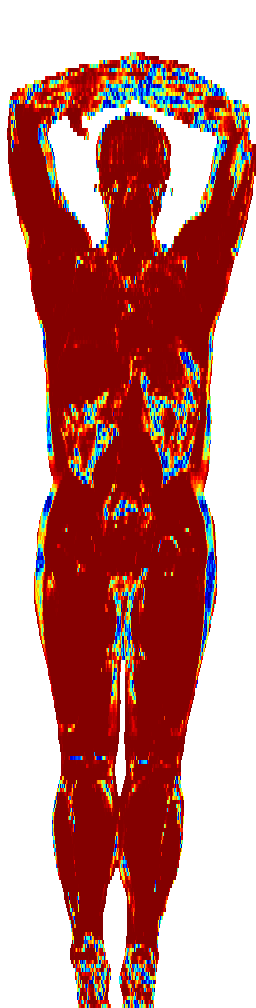 | 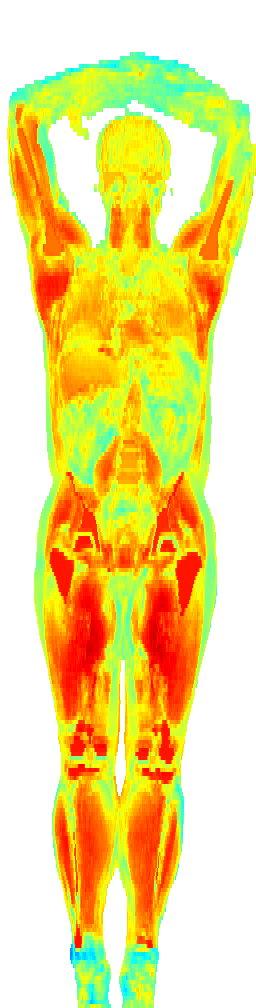 | 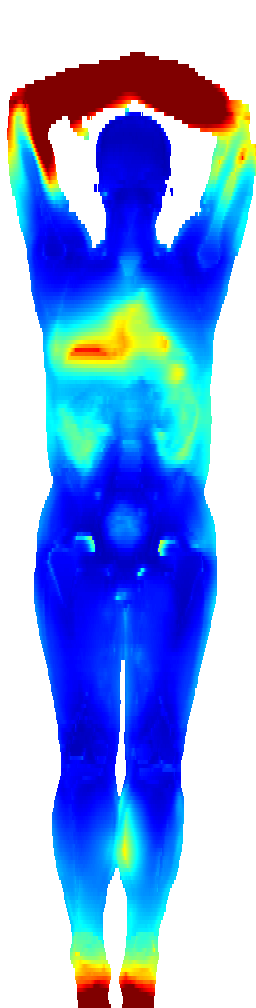 | 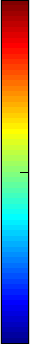 | 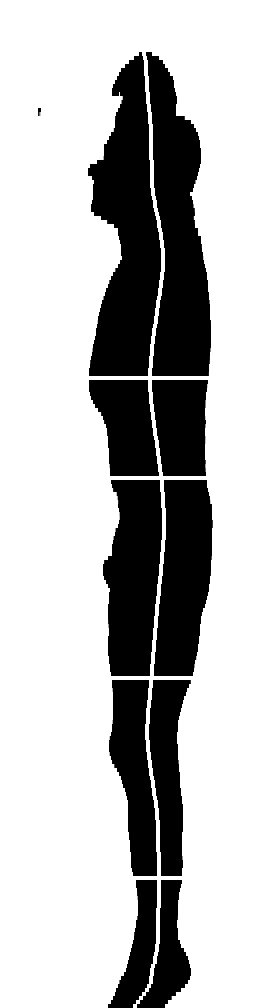 |
| 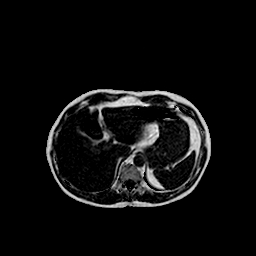 | 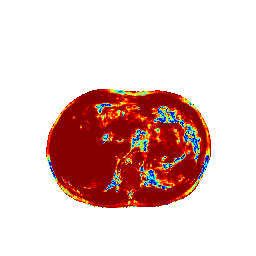 | 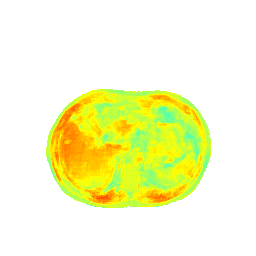 | 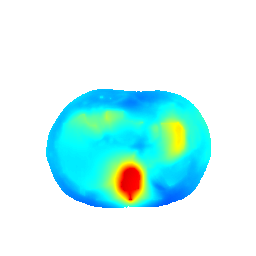 |  | |
| 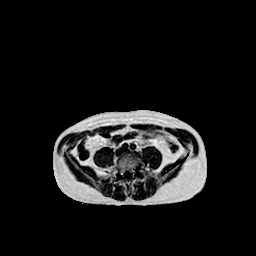 | 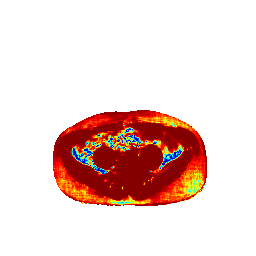 | 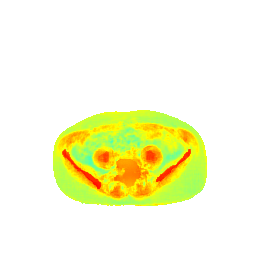 | 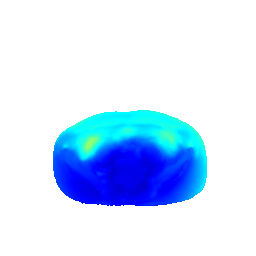 |  |  |
| 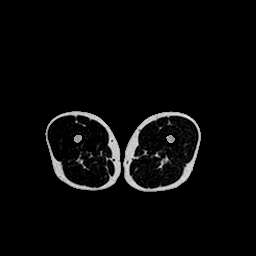 | 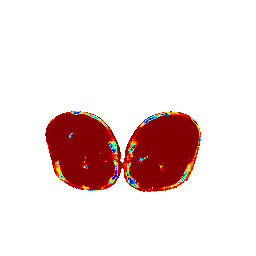 | 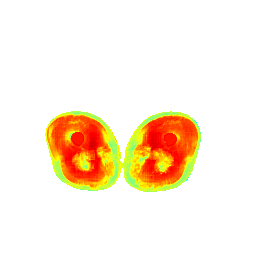 | 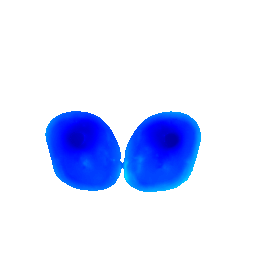 |  |  |
| 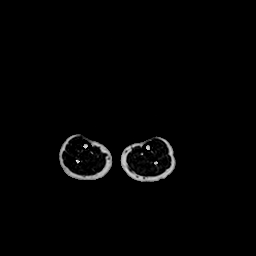 | 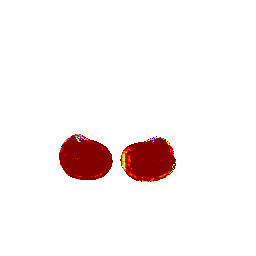 | 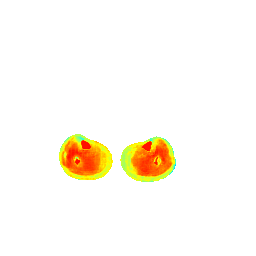 | 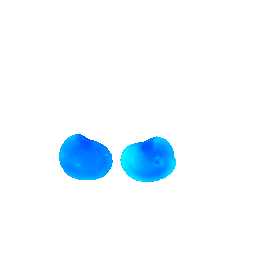 |  |  |
|  |  |  |  |  |  |

**S4 Fig. Imiomics analysis – local tissue volume vs. total lean mass for women.** Imiomics analysis of local tissue volume (from whole body MRI) vs. total lean mass (measured by DXA) for women. This figure gives detailed information to supplement the fourth subfigures from the left in Fig 2 in the main manuscript. The fat content values are between 0% (black) and 100% (white). The interval to which the colors in the colormap (shown to the right) is mapped to is shown within brackets for p-map, r-map and the displacement error as computed by inverse consistency. The position of the curved coronal and four axial slices are shown in the right panel. N=159.

| Fat | p map | r map | displacement error |  | |
| --- | --- | --- | --- | --- | --- |
| [0 100] | [1 0] (red sign.) | [-1 1] | [0 20] mm |  |  |
| 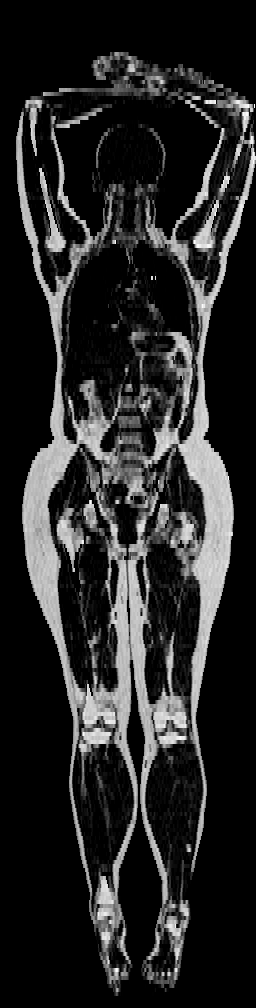 | 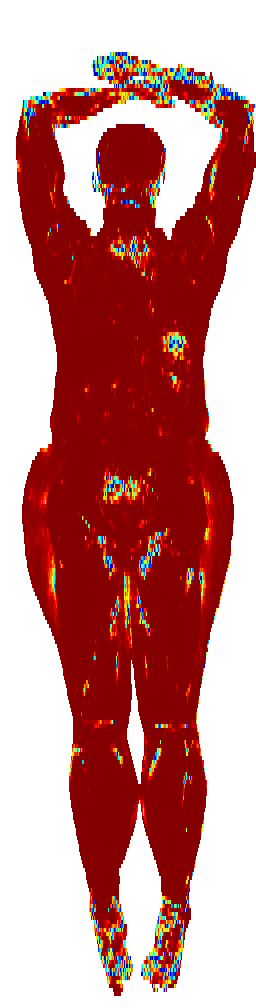 | 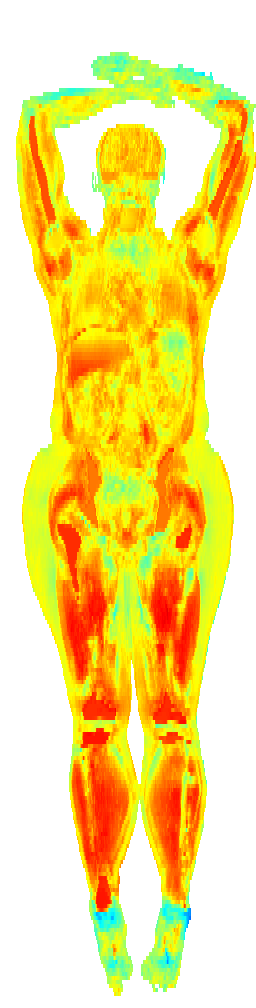 | 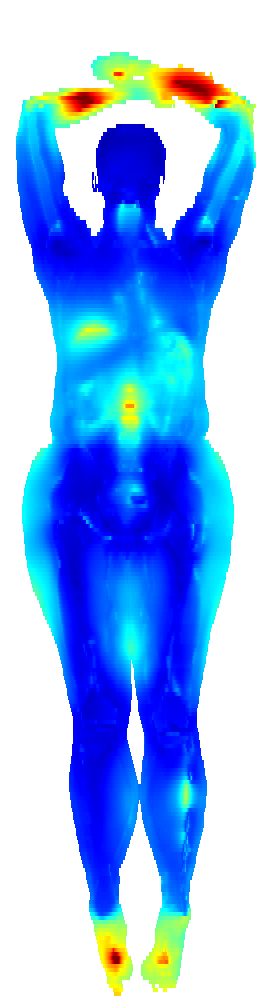 | 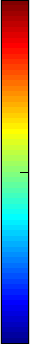 | 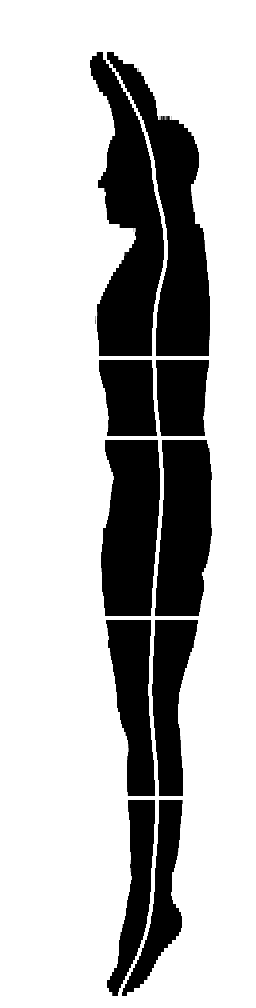 |
| 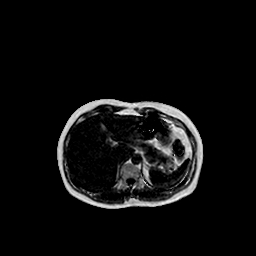 | 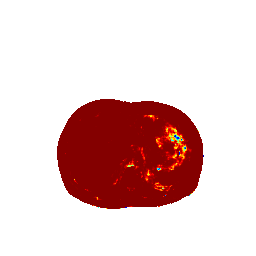 | 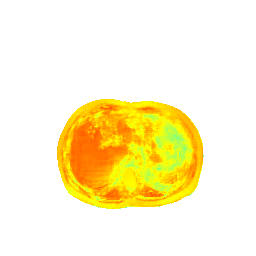 | 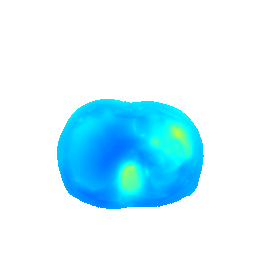 |  | |
| 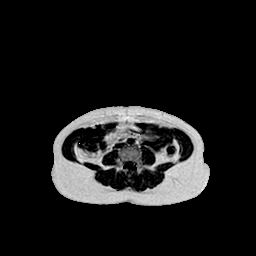 | 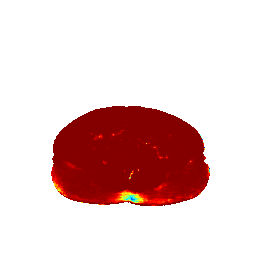 | 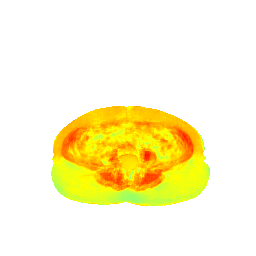 | 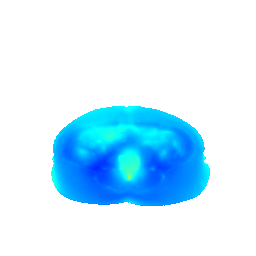 |  |  |
| 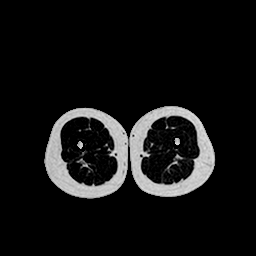 | 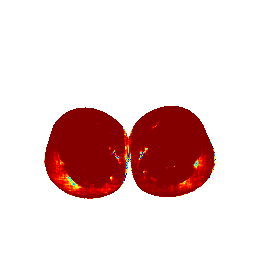 | 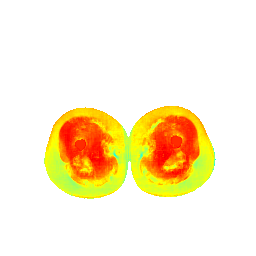 | 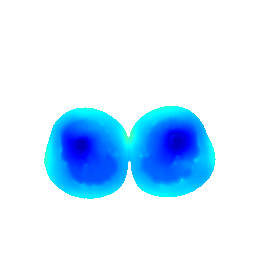 |  |  |
| 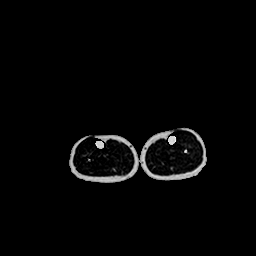 | 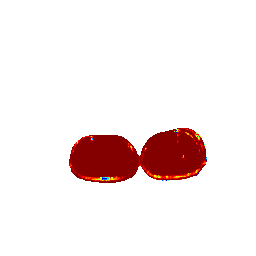 | 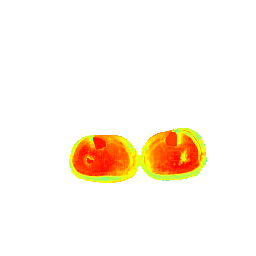 | 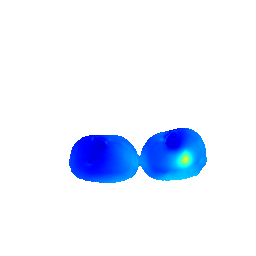 |  |  |
|  |  |  |  |  |  |

**S5 Fig. Imiomics analysis –local tissue volume vs. total lean mass for men.** Imiomics analysis of local tissue volume (from whole body MRI) vs. waist-hip-ratio for men. This figure gives detailed information to supplement the fifth subfigures from the left in Fig 2 in the main manuscript. The fat content values are between 0% (black) and 100% (white). The interval to which the colors in the colormap (shown to the right) is mapped to is shown within brackets for p-map, r-map and the displacement error as computed by inverse consistency. The position of the curved coronal and four axial slices are shown in the right panel. N=151.

| Fat | p map | r map | displacement error |  | |
| --- | --- | --- | --- | --- | --- |
| [0 100] | [1 0] (red sign.) | [-1 1] | [0 20] mm |  |  |
| 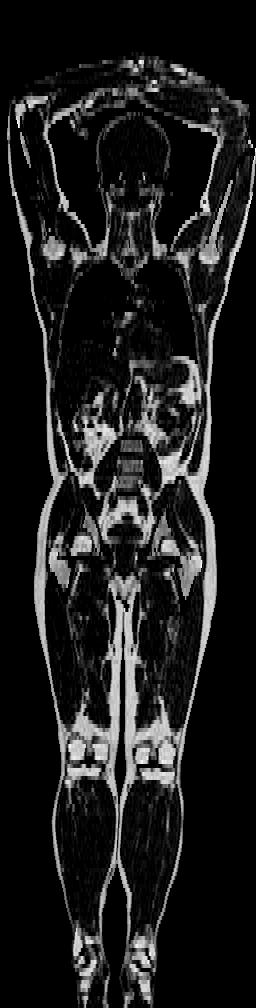 | 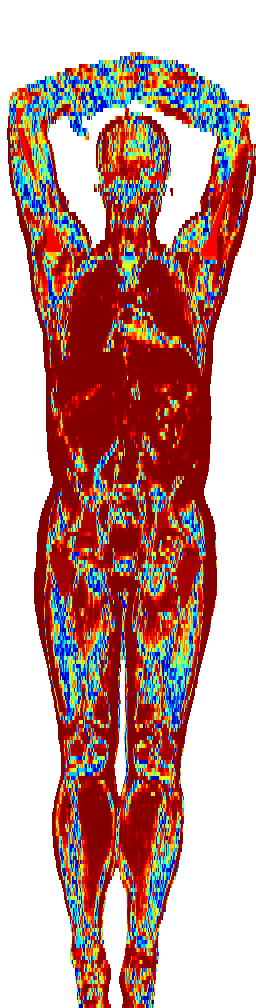 | 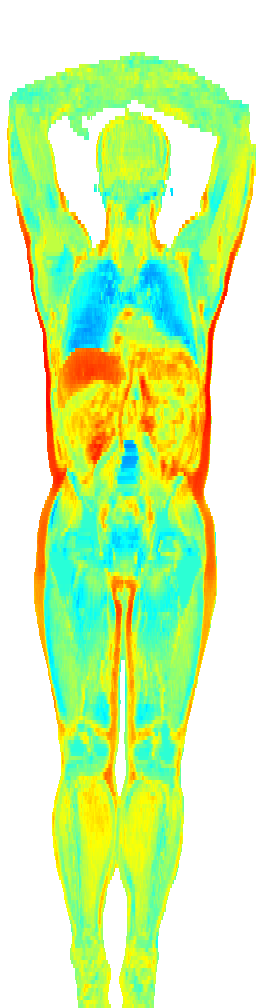 | 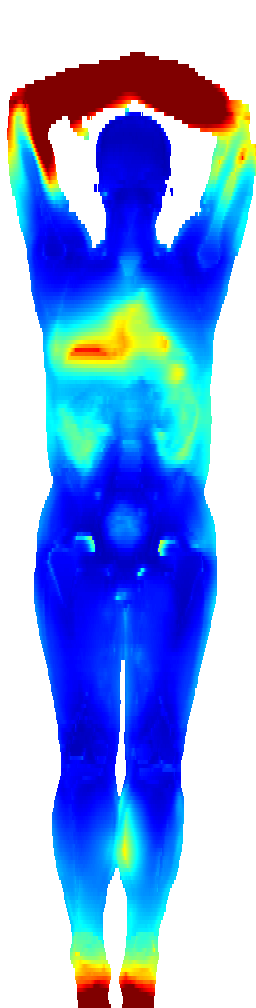 | 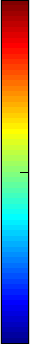 | 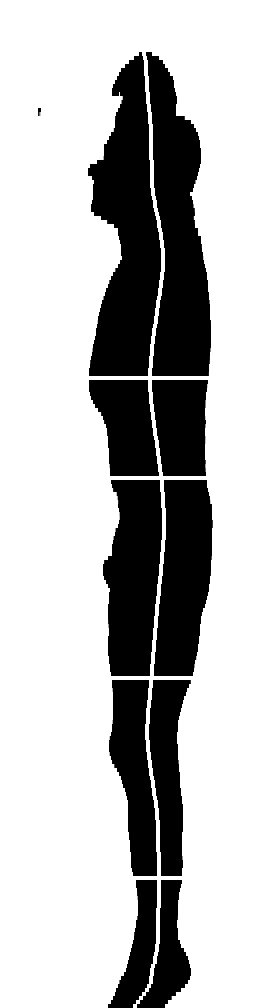 |
| 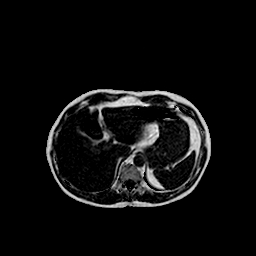 | 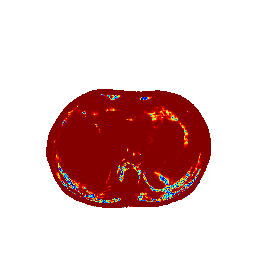 | 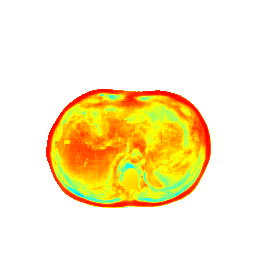 | 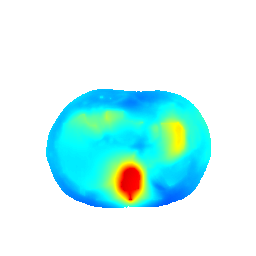 |  | |
| 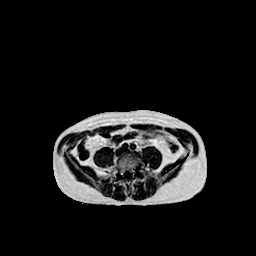 | 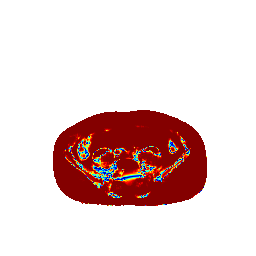 |  |  |  |  |
|  |  |  |  |  |  |
|  |  |  |  |  |  |
|  |  |  |  |  |  |

**S6 Fig. Imiomics analysis – local tissue volume vs. total lean mass for women.** Imiomics analysis of local tissue volume (from whole body MRI) vs. waist-hip-ratio for women. This figure gives detailed information to supplement the sixth subfigures from the left in Fig 2 in the main manuscript. The fat content values are between 0% (black) and 100% (white). The interval to which the colors in the colormap (shown to the right) is mapped to is shown within brackets for p-map, r-map and the displacement error as computed by inverse consistency. The position of the curved coronal and four axial slices are shown in the right panel. N=167.

| Fat | p map | r map | displacement error |  | |
| --- | --- | --- | --- | --- | --- |
| [0 100] | [1 0] (red sign.) | [-1 1] | [0 20] mm |  |  |
|  |  |  |  |  |  |
|  |  |  |  |  | |
|  |  |  |  |  |  |
|  |  |  |  |  |  |
|  |  |  |  |  |  |
|  |  |  |  |  |  |

**S7 Fig. Imiomics analysis – fat content vs. total fat mass for men.** Imiomics analysis of fat content (from whole body MRI) vs. total fat mass (measured by DXA) for men. This figure gives detailed information to supplement the leftmost subfigures in Fig 3 in the main manuscript. The fat content values are between 0% (black) and 100% (white). The interval to which the colors in the colormap (shown to the right) is mapped to is shown within brackets for p-map, r-map and the displacement error as computed by inverse consistency. The position of the curved coronal and four axial slices are shown in the right panel. N=151.

| Fat | p map | r map | displacement error |  | |
| --- | --- | --- | --- | --- | --- |
| [0 100] | [1 0] (red sign.) | [-1 1] | [0 20] mm |  |  |
|  |  |  |  |  |  |
|  |  |  |  |  | |
|  |  |  |  |  |  |
|  |  |  |  |  |  |
|  |  |  |  |  |  |
|  |  |  |  |  |  |

**S8 Fig. Imiomics analysis – fat content vs. total fat mass for women.** Imiomics analysis of local tissue volume (from whole body MRI) vs. total fat mass (measured by DXA) for women. This figure gives detailed information to supplement the second subfigures from the left in Fig 3 in the main manuscript. The fat content values are between 0% (black) and 100% (white). The interval to which the colors in the colormap (shown to the right) is mapped to is shown within brackets for p-map, r-map and the displacement error as computed by inverse consistency. The position of the curved coronal and four axial slices are shown in the right panel. N=156.

| Fat | p map | r map | displacement error |  | |
| --- | --- | --- | --- | --- | --- |
| [0 100] | [1 0] (red sign.) | [-1 1] | [0 20] mm |  |  |
|  |  |  |  |  |  |
|  |  |  |  |  | |
|  |  |  |  |  |  |
|  |  |  |  |  |  |
|  |  |  |  |  |  |
|  |  |  |  |  |  |

**S9 Fig. Imiomics analysis – fat content vs. total lean mass for men.** Imiomics analysis of fat content (from whole body MRI) vs. total lean mass (measured by DXA) for men. This figure gives detailed information to supplement the third subfigures from the left in Fig 3 in the main manuscript. The fat content values are between 0% (black) and 100% (white). The interval to which the colors in the colormap (shown to the right) is mapped to is shown within brackets for p-map, r-map and the displacement error as computed by inverse consistency. The position of the curved coronal and four axial slices are shown in the right panel. N=151.

| Fat | p map | r map | displacement error |  | |
| --- | --- | --- | --- | --- | --- |
| [0 100] | [1 0] (red sign.) | [-1 1] | [0 20] mm |  |  |
|  |  |  |  |  |  |
|  |  |  |  |  | |
|  |  |  |  |  |  |
|  |  |  |  |  |  |
|  |  |  |  |  |  |
|  |  |  |  |  |  |

**S10 Fig. Imiomics analysis – fat content vs. total lean mass for women.** Imiomics analysis of fat content (from whole body MRI) vs. total lean mass (measured by DXA) for women. This figure gives detailed information to supplement the fourth subfigures from the left in Fig 3 in the main manuscript. The fat content values are between 0% (black) and 100% (white). The interval to which the colors in the colormap (shown to the right) is mapped to is shown within brackets for p-map, r-map and the displacement error as computed by inverse consistency. The position of the curved coronal and four axial slices are shown in the right panel. N=159.

| Fat | p map | r map | displacement error |  | |
| --- | --- | --- | --- | --- | --- |
| [0 100] | [1 0] (red sign.) | [-1 1] | [0 20] mm |  |  |
|  |  |  |  |  |  |
|  |  |  |  |  | |
|  |  |  |  |  |  |
|  |  |  |  |  |  |
|  |  |  |  |  |  |
|  |  |  |  |  |  |

**S11 Fig. Imiomics analysis – fat content vs. total lean mass for men.** Imiomics analysis of fat content (from whole body MRI) vs. waist-hip-ratio for men. This figure gives detailed information to supplement the fifth subfigures from the left in Fig 3 in the main manuscript. The fat content values are between 0% (black) and 100% (white). The interval to which the colors in the colormap (shown to the right) is mapped to is shown within brackets for p-map, r-map and the displacement error as computed by inverse consistency. The position of the curved coronal and four axial slices are shown in the right panel. N=151.

| Fat | p map | r map | displacement error |  | |
| --- | --- | --- | --- | --- | --- |
| [0 100] | [1 0] (red sign.) | [-1 1] | [0 20] mm |  |  |
|  |  |  |  |  |  |
|  |  |  |  |  | |
|  |  |  |  |  |  |
|  |  |  |  |  |  |
|  |  |  |  |  |  |
|  |  |  |  |  |  |

**S12 Fig. Imiomics analysis – fat content vs. total lean mass for women.** Imiomics analysis of fat content (from whole body MRI) vs. waist-hip-ratio for women. This figure gives detailed information to supplement the sixth subfigures from the left in Fig 3 in the main manuscript. The fat content values are between 0% (black) and 100% (white). The interval to which the colors in the colormap (shown to the right) is mapped to is shown within brackets for p-map, r-map and the displacement error as computed by inverse consistency. The position of the curved coronal and four axial slices are shown in the right panel. N=167.

| Fat | p map | r map | displacement error |  | |
| --- | --- | --- | --- | --- | --- |
| [0 100] | [1 0] (red sign.) | [-1 1] | [0 20] mm |  |  |
|  |  |  |  |  |  |
|  |  |  |  |  | |
|  |  |  |  |  |  |
|  |  |  |  |  |  |
|  |  |  |  |  |  |
|  |  |  |  |  |  |
